# Adverse events associated with herbal medicine products reported in the Korea Adverse Event Reporting System from 2012 to 2021

**DOI:** 10.3389/fphar.2024.1378208

**Published:** 2024-10-21

**Authors:** Yujin Choi, Hyeun-Kyoo Shin

**Affiliations:** KM Science Research Division, Korea Institute of Oriental Medicine, Daejeon, Republic of Korea

**Keywords:** adverse drug reaction reporting systems, adverse effects, drug-related side effects and adverse reactions, herbal medicine, KAERS DB, pharmacovigilance

## Abstract

**Introduction:**

Systematic collection of diverse adverse events during herbal medicine administration is crucial. The Korea Adverse Event Reporting System (KAERS) compiles spontaneously reported adverse event data for medicinal products including herbal medicines. This study focused on extracting and analyzing adverse event data specifically related to herbal medicine products from the KAERS database.

**Methods:**

Individual case safety reports (ICSRs) encompassing 84 types of herbal medicine products, identified by item codes from 2012 to 2021, were extracted from the KAERS database. Descriptive statistics were employed to analyze the characteristics of the extracted reports, and adverse event information was systematically categorized and analyzed based on the MedDRA System Organ Class and preferred term classification.

**Results:**

In total, 1,054 ICSRs were extracted, with some documenting multiple adverse events in a single ICSR, resulting in 1,629 extracted adverse events. When categorized by the MedDRA System Organ Class, gastrointestinal disorders were the most prevalent (28.7%), followed by skin and subcutaneous tissue disorders (20.1%). Based on the preferred terms, the most frequently reported adverse events were diarrhea (5.8%), urticaria (5.3%), pruritus (4.7%), rash (4.4%), and abdominal discomfort (4.2%). The most frequently reported herbal medicines were Bangpungtongseong-san (297 cases), Kyeongok-go (144 cases), and Eunkyo-san (108 cases).

**Conclusion:**

Spontaneously reported adverse events associated with herbal medicine products were systematically documented using the KAERS database. This study, which focused on voluntarily reported adverse reactions, underscores the need for additional research to estimate the incidence rate of adverse events and assess causality.

## 1 Introduction

Herbal medicine occupies a prominent position in traditional and complementary medical practices, encompassing Traditional East Asian Medicine (TEAM) and Ayurveda, and has been extensively employed to address various health conditions (World Health Organization, 2019). Despite the global perception that herbal medicines are safe owing to their natural origin and historical use, vigilant monitoring is necessary for adverse effects akin to other pharmaceuticals (Zhang et al., 2012; Choudhury et al., 2023). As a strategy for advancing traditional medicine, the World Health Organization (WHO) underscored the importance of providing evidence regarding the safety of herbal medicines through pharmacovigilance monitoring systems (World Health Organization, 2013). Countries affiliated with TEAM, such as China, Korea, Japan, and Taiwan, have implemented regulations concerning herbal medicines and monitored adverse events under pharmacovigilance systems for both synthetic medicines and herbal products (Kimura et al., 2011; Park et al., 2012; Zhang et al., 2012).

In Korea, the Korea Adverse Event Reporting System (KAERS) was established to systematically collect adverse reactions associated with pharmaceutical use (Shin et al., 2014). Initiated in 1988 by the Korea Ministry of Food and Drug Safety (MFDS) and jointly overseen by the Korea Institute of Drug Safety and Risk Management since 2012, KAERS facilitates the reporting and management of adverse cases through a computerized system. Reports of adverse drug reactions are accepted from pharmaceutical companies, healthcare professionals, and consumers. Since its inception in 1989 with 13 reported cases, this reporting system was stabilized in the 2000s. From 2010 onwards, there was a significant increase in reported cases, leading to the successful establishment of a pharmacovigilance system (Kang et al., 2017). In 2020, there were 259,089 reported cases, and in 2021, with adverse reactions to COVID-19 vaccines, the total reached 539,441 cases (Ministry of Food and Drug Safety, 2023). Adverse drug reaction cases for licensed herbal medicine products (HMPs) authorized by the Korea MFDS can also be reported through the KAERS (Woo et al., 2014). Since 2014, there have been 27 regional pharmacovigilance centers to collect adverse event data, and in 2020, an additional regional pharmacovigilance center specifically focused on HMPs was established, making it a total of 28 centers (Korea Institute of Drug Safety and Risk Management, 2024). However, it is important to note that the KAERS, like other spontaneous reporting systems, has inherent limitations. Only observed adverse events are registered, resulting in underreporting and reporting bias, which requires proper interpretation of data from the KAERS and other spontaneous reporting systems (Noguchi et al., 2021).

Globally, adverse events associated with herbal medicines have been documented in spontaneous adverse event databases. The WHO receives reports on adverse reactions to herbal medicines and establishes guidelines for monitoring their safety (World Health Organization, 2004). An expert survey revealed that countries throughout Asia, Europe, Africa, and America include herbal medicines in their adverse event spontaneous reporting systems with varying approaches to managing different types of herbal medicines (Kim et al., 2021b). Several studies based on these pharmacovigilance system databases have been published. For instance, a study has focused on hepatobiliary disorders linked to herbal medicine, utilizing the WHO global database Vigibase (van Hunsel et al., 2019). Other studies have conducted comparative analyses of adverse event reports between Chinese and Western medicines, relying on the Chinese Spontaneous Reporting Database (Wei et al., 2022). In Japan, studies have estimated the incidence of adverse drug reactions related to Kampo medicine based on data from the Japanese Adverse Drug Event Database (Shimada et al., 2019; Arai et al., 2020; Uneda et al., 2024). Additionally, research has been conducted on adverse events associated with herbal medicines using Taiwan’s Adverse Drug Reaction Reporting System for Herbal Medicine (Chang et al., 2021).

Despite this wealth of information, there is currently no literature on adverse event reports associated with herbal medicines in Korea based on the Korean spontaneous adverse event database. According to a study on adverse events attributed to traditional Korean medicine practices, only eight adverse events related to HMPs were reported to Korea’s pharmacovigilance system before 2010 (Shin et al., 2013) Since the stabilization of Korea’s pharmacovigilance system, there has been no comprehensive research analyzing adverse event related to HMPs using the KAERS database. Given the significant use of TEAM, including herbal medicines, within the national healthcare system in Korea, this study aimed to fill this gap by focusing on the extraction and analysis of adverse event data specifically related to HMPs from the KAERS database from 2012 to 2021.

## 2 Materials and methods

### 2.1 Data sources

The Korea Institute of Drug Safety and Risk Management (KIDS) is currently developing a KAERS database by standardizing codes for pharmaceutical and adverse event information and creating a format suitable for analytical procedures. We acquired data from the KIDS KAERS database, specifically focusing on individual case safety reports (ICSRs) encompassing 84 types of HMPs from 2012 to 2021 (Data No. 2205A0042).

Each ICSR was comprised of basic information, adverse event information, pharmaceutical information, and causality assessment results. Basic information included the report year, report type (voluntary reporting, reporting in trials/research), original reporter (doctors, pharmacists, other medical professionals, and consumers), reporter (pharmaceutical company, regulatory authority, hospital/pharmacies, regional drug safety center, and consumers), and demographic information of the patients (age and sex). Adverse events were coded using the Medical Dictionary for Regulatory Activities (MedDRA) lowest-level term. The reported adverse events were categorized into System Organ Classes (SOCs) and preferred terms (PTs) using MedDRA version 26.0. Additionally, the start date and end date of the adverse event, and outcomes of adverse events (recovered or resolved, recovering or resolving, not recovered or not resolved, recovered or resolved with sequelae, or fatal) were also recorded. For each adverse event, it was also reported whether it corresponded to serious adverse events, including death, life-threatening conditions, initial or prolonged hospitalization, and other important medical events. Pharmaceutical information included the type of medicine (suspected drug, concomitant drug, or interacting drug), product codes, and ingredient codes provided by the Korean MFDS. Details regarding actions taken with the medicine (drug withdrawn, dose reduced, dose not changed, or not applicable) and the start and end dates of administration were also included. Causality assessment information was provided for each adverse event and drug combination, with causality categorized as certain, probable/likely, possible, unlikely, conditional/unclassified, or unassessable/unclassifiable.

### 2.2 Herbal medicine products

This study focused on 84 frequently used HMPs selected from among the 134 licensed HMPs currently produced in Korea (Korea Pharmaceutical and Bio-Pharma Manufacturers Association, 2023). These 84 HMPs were chosen based on four criteria: national health insurance coverage, high production volume, classification as basic herbal medicine formula in standard textbooks, and frequency of use in TEAM. We included only licensed HMPs approved by the Korean MFDS for use as pharmaceuticals, as the KAERS databases only collects ICSRS for approved, licensed drugs. Consequently, individualized herbal preparations from Korean medicine clinics of hospitals, as well as herbal dietary supplements or functional foods, were excluded from this study. The complete list of selected HMPs is provided in Supplementary Table S1.

### 2.3 Inclusion criteria for analysis

This study included ICSRs involving at least one HMP from a list of 84 HMPs in Korea. ICSRs involving HMPs as suspected drugs and those involving HMPs as concomitant or interacting drugs were also included. Each ICSR included multiple adverse events and drugs. Regarding adverse event information, all reported adverse events in the included ICSRs were analyzed. Regarding pharmaceutical information, all 84 HMPs reported in the included ICSRs were analyzed. The causality assessment results were included for the combination of adverse events and 84 HMPs.

### 2.4 Critical adverse events

Based on previous studies on adverse events associated with herbal medicine (Arai et al., 2020; Chino et al., 2020), critical adverse events were defined as hepatobiliary adverse drug reactions, pulmonary adverse drug reactions, anaphylactic responses, and pseudoaldosteronism. To investigate adverse events in these four areas, for hepatobiliary adverse drug reaction, we identified adverse events that fell under the SOC of “Hepatobiliary disorders” (van Hunsel et al., 2019) or the High-Level Group Term (HLGT) of “Hepatobiliary investigations.” For pulmonary adverse drug reactions, we included cases where the reported SOC was “Respiratory, thoracic and mediastinal disorders” or the High-Level Term (HLT) was “Lower respiratory tract and lung infections.” To exclude upper respiratory tract disorders, we excluded cases where the HLGT was “Upper respiratory tract disorders (excl infections)” or the HLT was “Nasal congestion and inflammations,” “Nasal disorders NEC,” “Pharyngeal disorders (excl infections and neoplasms),” or “Upper respiratory tract signs and symptoms.” For anaphylactic responses, we extracted cases where the reported HLT was “Anaphylactic and anaphylactoid responses.” For pseudoaldosteronism, we extracted cases in which the PT showed pseudoaldosteronism, blood potassium decrease, or hypokalemia (Ishida et al., 2020; Uneda et al., 2024).

### 2.5 Data analysis

The ICSRs extracted from the KAERS database are provided in tables in ASCII format containing basic information, adverse event information, pharmaceutical information, and causality assessment results. Each table includes a randomly generated reporting number called “KARES_NO” as a connecting key across tables. Data were processed using the tidyverse package (Wickham et al., 2019) in R (version 4.2.3) (R Core Team, 2013), and frequencies and percentages were presented for each category. To compare the demographics of safety reports between serious adverse events and non-serious adverse events, odds ratios and 95% confidence intervals were calculated for serious adverse event reporting versus not serious adverse events between two groups (≥65 years vs. <64 years, and female vs. male) (Noguchi and Yoshimura, 2024). Additionally, for the 15 most frequently reported adverse events related to HMPs, a disproportionality analysis was conducted to compare the number of reported adverse events associated with HMPs and the total number of corresponding adverse events reported in Korea during the study period. This analysis method is used for signal detection purposes in spontaneously reported adverse events, and proportional reporting ratio (PRR), reporting odds ratio (ROR), and Information component (IC) were calculated for each adverse event (Noguchi et al., 2021).

## 3 Results

### 3.1 Basic information of the ICSRs

**FIGURE 1:**
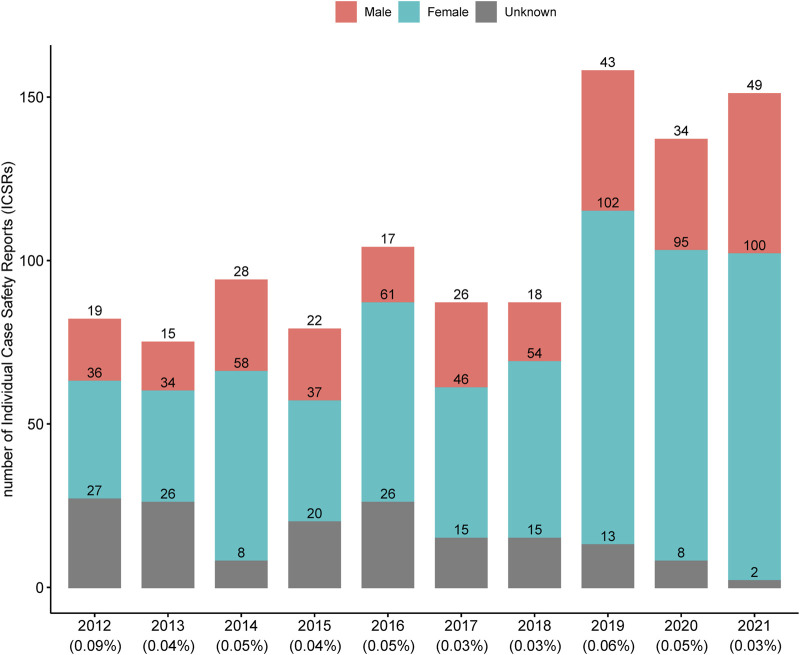
Annual number of individual case safety reports associated with licensed herbal medicine products in Korea. The percentages below each year represent the proportion of individual case safety reports associated with licensed herbal medicine products out of the total individual case safety reports.

A total of 1,054 ICSRs related to 84 HMPs were extracted from the KAERS database for the period from 2012 to 2021 (10 years). The annual number of ICSRs from 2012 to 2021 is presented in Figure 1 along with the percentage of total ICSRs reported annually by the MFDS (Korea Ministry of Food and Drug Safety, 2023). Until 2018, the number of ICSRs related to HMPs was generally less than 100. However, after 2019, there was a noticeable increase, exceeding 137 cases. The percentage of ICSRs associated with HMPs among the total ICSRs remained relatively consistent, ranging from 0.03% to 0.09%.

Table 1 summarizes basic information of the 1,054 ICSRs, including details about the reporters and demographics of patients experiencing adverse drug reactions. The original reporter referred to a person who first became aware of an adverse event and reported it. Pharmacists accounted for the highest percentage (44.6%), followed by consumers (28.2%) and doctors (13.1%). Regarding the demographics of patients experiencing adverse reactions, 56.5% were aged 19–65 years, and the number of reports by women was more than twice than by men. Among the 1,054 ICSRs, 580 (55%) involved the consumption of HMP alone, whereas 474 (45%) involved the concurrent use of other medications along with HMP.

**TABLE 1:** Basic information of 1,054 safety reports associated with herbal medicine products.

Information of reports	n (%)
Original reporter	
Doctors, dentists, Korean medicine doctors	178 (16.9)
Pharmacists, Korean medicine pharmacists	470 (44.6)
Other medical professionals	65 (6.2)
Consumers, non-medical professionals	297 (28.2)
Unknown	44 (4.2)
Reporter	
Pharmaceutical company	377 (35.8)
Medical experts (e.g., hospitals, pharmacies)	102 (9.7)
Regional pharmacovigilance center	469 (44.5)
Others (e.g., distributors)	42 (4.0)
Patients, consumers	64 (6.1)
Report type	
Voluntary reporting	950 (90.1)
Reporting in trials/research	78 (7.4)
Others	24 (2.3)
Unknown	2 (0.2)
Patient demographics^a^	
Age at the time of occurrence	
0–27 days	2 (0.3)
28 days to 1 year	4 (0.5)
2–11 years	6 (0.8)
12–18 years	10 (1.3)
19–64 years	596 (76.9)
65 years ∼	157 (20.3)
Sex	
Male	271 (30.3)
Female	623 (69.7)
Drugs	
Herbal medicine only	580 (55.0)
Combination with other medications	474 (45.0)

^a^ Regarding age, 279 patients had missing data; regarding sex, 160 patients had missing data.

### 3.2 Characteristics of reported adverse events

Because some of the 1,054 ICSRs included multiple adverse events, 1,629 adverse events were included in the analysis. The classification of the 1,629 reported adverse events by SOC is presented in Table 2. Gastrointestinal disorders were the most frequently reported (28.7%), followed by skin and subcutaneous tissue disorders (20.1%) and nervous system disorders (11.4%). The number of adverse events at the HLGT level for each SOC is shown in Supplementary Table S2. Based on the PT in MedDRA, Table 3 presents a list of the 15 most frequently reported adverse events and the results of the disproportionality analysis. Notably, within gastrointestinal disorders, diarrhea, abdominal discomfort, dyspepsia, nausea, abdominal pain, vomiting, dry mouth, and constipation were commonly reported. Urticaria, pruritus, rash, and angioedema are frequently reported skin and subcutaneous tissue disorders. Insomnia is notable in the subcategories of psychiatric disorders, whereas dizziness, headaches, and somnolence are frequently reported in nervous system disorders. According to the results of the disproportionality analysis, adverse events that were reported more frequently with HMPs compared to all other drugs (PRR ≥2, χ^2^ ≥ 4, ROR ≥2, and IC_025_ > 0) included abdominal discomfort, insomnia, and palpitations (Table 3).

**TABLE 2:** Characteristics of 1,629 adverse events.

Adverse events classified by system organ class	n (%)
Gastrointestinal disorders	467 (28.7)
Skin and subcutaneous tissue disorders	327 (20.1)
Nervous system disorders	185 (11.4)
General disorders and administration site conditions	146 (9.0)
Psychiatric disorders	110 (6.8)
Investigations^a^	81 (5.0)
Respiratory, thoracic, and mediastinal disorders	64 (3.9)
Cardiac disorders	44 (2.7)
Eye disorders	35 (2.1)
Musculoskeletal and connective tissue disorders	32 (2.0)
Infections and infestations	23 (1.4)
Vascular disorders	23 (1.4)
Immune system disorders	19 (1.2)
Injury, poisoning, and procedural complications	18 (1.1)
Metabolism and nutrition disorders	17 (1.0)
Renal and urinary disorders	13 (0.8)
Reproductive system and breast disorders	8 (0.5)
Ear and labyrinth disorders	6 (0.4)
Hepatobiliary disorders	5 (0.3)
Blood and lymphatic system disorders	3 (0.2)
Product issues	3 (0.2)
Duration of adverse events	
−1 day	110 (6.8)
2–5 days	69 (4.2)
6–10 days	40 (2.5)
11–50 days	55 (3.4)
50 days ∼	8 (0.5)
(Missing)	1,347 (82.7)
Results of adverse events	
Recovered or resolved	569 (34.9)
Recovering or resolving	108 (6.6)
Not Recovered or not resolved	75 (4.6)
Recovered or resolved with sequelae	59 (3.6)
Fetal	9 (0.6)
Unknown	809 (49.7)

^a^ 81 adverse events under the SOC, of Investigation included hepatobiliary investigations (48 cases); physical examination and organ system status topics (15 cases); cardiac and vascular investigations (9 cases); metabolic, nutritional, and blood gas investigations (4 cases); renal and urinary tract investigations and urinalyses (3 cases); and neurological, special senses, and psychiatric investigations (2 cases).

**TABLE 3:** The 15 most frequently reported adverse events and results of disproportionality analysis.

System organ class	Adverse event (preferred Term)	n (%)	PRR	ROR	IC_025_	χ^2^
Gastrointestinal disorders	Diarrhea	95 (5.8)	1.82	1.87	0.15	35.43
Skin and subcutaneous tissue disorders	Urticaria	87 (5.3)	0.69	0.67	−1.25	12.63
Skin and subcutaneous tissue disorders	Pruritus	77 (4.7)	0.56	0.54	−1.60	28.82
Skin and subcutaneous tissue disorders	Rash	72 (4.4)	0.69	0.68	−1.32	10.02
Gastrointestinal disorders	Abdominal discomfort	69 (4.2)	5.10	5.28	1.45	224.21
Psychiatric disorders	Insomnia	67 (4.1)	5.82	6.03	1.61	263.60
Nervous system disorders	Dizziness	54 (3.3)	0.36	0.34	−2.37	66.62
Gastrointestinal disorders	Dyspepsia	51 (3.1)	1.31	1.32	−0.56	3.42
Nervous system disorders	Headache	51 (3.1)	0.47	0.45	−2.01	32.01
Gastrointestinal disorders	Nausea	49 (3.0)	0.18	0.16	−3.37	210.71
Gastrointestinal disorders	Abdominal pain	43 (2.6)	1.40	1.41	−0.54	4.68
Gastrointestinal disorders	Vomiting	43 (2.6)	0.35	0.33	−2.52	56.46
Cardiac disorders	Palpitations	37 (2.3)	3.10	3.15	0.46	50.88
Gastrointestinal disorders	Dry mouth	32 (2.0)	2.30	2.32	−0.03	22.35
Gastrointestinal disorders	Constipation	30 (1.8)	1.24	1.25	−0.90	1.20

ALT, alanine aminotransferase; AST, aspartate aminotransferase.

The duration of the adverse events was calculated by subtracting the end date of the adverse event from the start date. However, approximately 82.7% of the duration data was missing. Among the available data, the most frequently reported duration was 1 day. The results of adverse events were also reported, with approximately half (49.7%) categorized as unknown. Following this, 34.9% were reported as recovered, and 6.6% were in the process of recovery.

### 3.3 Serious adverse events

Serious adverse events, including death, life-threatening conditions, initial or prolonged hospitalization, and other important medical events, were individually evaluated for each reported adverse event. Out of the 1,054 ICSRs, 48 (4.6%) included one or more serious adverse events (four ICSRs were identified with duplicate labels spanning two serious adverse event categories). Further, among the 1,629 adverse events, 99 (6.1%) were identified as serious adverse events (Table 4). Two cases of death were reported, with causality categorized as unknown or unlikely. Regarding life-threatening conditions, three cases of dyspnea, nausea, and dizziness have been reported, all of which resulted in recovery. Thirty-one cases of initial or prolonged hospitalization have been reported. Among the adverse events associated with hospitalization (51 events in total, considering the multiple events reported in one ICSR with hospitalization), increased aspartate aminotransferase and alanine aminotransferase levels were the most frequent (7 cases each), followed by dyspnea (3 cases), liver function test abnormal (2 cases), nausea (2 cases), and rash (2 cases). Sixteen cases of other important medical events were reported, and among 43 related events, dyspnea (5 cases), angioedema (4 cases), urticaria (4 cases), anaphylactic reaction (3 cases), chest discomfort (2 cases), and dizziness (2 cases) were frequently reported (Supplementary Table S3).

**TABLE 4:** Characteristics of 48 safety reports including 99 adverse events reported as serious adverse events.

Patient demographics of 48 ICSRs^a^	n (%)
Age at the time of occurrence	
0–27 days	0 (0.0)
28 days to 1 year	1 (2.2)
2–11 years	0 (0.0)
12–18 years	1 (2.2)
19–64 years	34 (75.6)
65 years ∼	9 (20.0)
Sex	
Male	17 (35.4)
Female	31 (64.6)
Category of serious adverse event in 48 ICSRs/99 adverse events^b^	
Death	2 (4.2)/8 (8.1)
Life-threatening	3 (6.3)/3 (3.0)
Hospitalization (initial or prolonged)	31 (64.6)/51 (51.5)
Disability or permanent damage	0 (0.0)/0 (0.0)
Congenital anomaly/birth defect	0 (0.0)/0 (0.0)
Important medical events	16 (33.3)/43 (43.4)
99 adverse events classified by System Organ Class	
Skin and subcutaneous tissue disorders	19 (19.2)
Investigations	17 (17.2)
Respiratory, thoracic and mediastinal disorders	12 (12.1)
General disorders and administration site conditions	10 (10.1)
Gastrointestinal disorders	9 (9.1)
Nervous system disorders	6 (6.1)
Eye disorders	4 (4.0)
Immune system disorders	4 (4.0)
Infections and infestations	4 (4.0)
Hepatobiliary disorders	3 (3.0)
Musculoskeletal and connective tissue disorders	3 (3.0)
Metabolism and nutrition disorders	2 (2.0)
Psychiatric disorders	2 (2.0)
Blood and lymphatic system disorders	1 (1.0)
Cardiac disorders	1 (1.0)
Injury, poisoning and procedural complications	1 (1.0)
Vascular disorders	1 (1.0)

^a^ Three patients had missing age data.

^b^ An ICSR, and an adverse event can be classified under more than two serious adverse event categories.

The age and gender distribution of ICSRs between reports including serious adverse events and the other reports were compared (Supplementary Table S4). There was no significant difference in the odds of reporting serious adverse events based on age (≥65 years vs. <64 years, odds ratio [OR] 0.98, 95% confidence interval [CI] 0.46–2.09) or gender (female vs. male, OR 0.78, 95% CI 0.43–1.44).

### 3.4 Herbal medicine products and associated adverse events

Among the 1,054 ICSRs, some included multiple HMPs, resulting in 1,105 reported instances of HMPs. Among these, 913 cases (82.6%) were reported as suspected medicines and 192 cases (17.4%) were reported as concomitant medicines. None of the HMPs were classified as an interacting drug (Table 5). Duration of use was calculated by subtracting the end date of administration from the start date. The majority (68.1%) had missing data, and 145 cases (13.1%) were recorded for a duration of 1 day or less. Regarding the action taken for HMPs, drug withdrawal was the most common, accounting for 494 cases (44.7%), and cases where the action taken was not reported amounted to 357 cases (32.3%).

**TABLE 5:** Characteristics of 1,105 reported licensed herbal medicine products.

Causality of each drug	n (%)
Suspected drug	913 (82.6)
Concomitant drug	192 (17.4)
Interacting drug	0 (0.0)
Administration period	
up to 1 day	145 (13.1)
2–5 days	94 (8.5)
6–10 days	26 (2.4)
11–50 days	70 (6.3)
50 days ∼	18 (1.6)
(Missing)	752 (68.1)
Action taken	
Drug withdrawn	494 (44.7)
Dose reduced	38 (3.4)
Dose not changed	82 (7.4)
Unknown	45 (4.1)
Not applicable	89 (8.1)
(Missing)	357 (32.3)

The most frequently reported HMPs were Bangpungtongseong-san (297 cases, 26.9%), Kyeongok-go (144 cases, 13.0%), and Eunkyo-san (108 cases, 9.8%). The compositions of these HMPs are provided in Supplementary Table S5. Table 6 lists the 15 most frequently reported HMPs and their associated ICSR numbers. Additionally, the production ranking of each HMP among the 134 HMPs in Korea is provided for reference.

**TABLE 6:** The 15 most frequently reported licensed herbal medicine products and their production amount ranking.

Licensed herbal medicine products^a^	n (%)	Production amount ranking^b^
Bangpungtongseong-san (Fangfengtongsheng-san, Bofutsusho-san)	297 (26.9)	6
Kyeongok-go (Qiongyu-gao, Keigyoku-kou)	144 (13.0)	2
Eunkyo-san (Yinqiao-san, Gingyo-san)	108 (9.8)	10
Uhwangchungsim-won (Niuhuangqingxin-whan, Goouseishin-gen)	62 (5.6)	1
Galgeun-tang (Gegen-tang, Kakkon-to)	52 (4.7)	8
Ssanghwa-tang (Shuanghe-tang, Souwa-to)	43 (3.9)	5
Bangkeehwangkee-tang (Fangjihuangqi-tang, Boiogi-to)	41 (3.7)	11
Cheonwangbosim-dan (Tianwangbuxin-dan, Tennouhoshin-tan)	38 (3.4)	7
Gongjin-dan (Gongchen-dan)	36 (3.3)	3
Socheongryong-tang (Xiaoqinglong-tang, Shoseiryu-to)	30 (2.7)	12
Banhasasim-tang (Banxiaxiexin-tang, Hangeshashin-to)	24 (2.2)	9
Jakyak gamcho-tang (Shaoyaogancao-tang, Shakuyakukanzo-to)	22 (2.0)	-
Ojeok-san (Wuji-san, Goshaku-san)	16 (1.4)	-
Sogunjung-tang (Xiaojianzhong-tang, Shokenchu-to)	15 (1.4)	-
Maekmundong-tang (Maimendong-tang, Bakumondo-to)	14 (1.3)	13

^a^ Herbal medicines with Chinese or Japanese names in parentheses. The compositions of these herbal medicines are provided in Supplementary Table S5.

^b^ Ranking of 134 licensed herbal medicine products by production amount in Korea (2021).

In Bangpungtongseong-san, the commonly reported adverse events included insomnia (n = 39), abdominal discomfort (n = 34), headache (n = 27), diarrhea (n = 23), palpitations (n = 21), dry mouth (n = 19), and abdominal pain (n = 16). In Kyeongok-go, the most frequently reported adverse events were diarrhea (n = 31), abdominal discomfort (n = 16), urticaria (n = 13), dyspepsia (n = 12), abdominal pain (n = 11), and rash (n = 11). Commonly reported adverse events of Eunkyo-san included urticaria (n = 12), pruritus (n = 20), rash (n = 20), angioedema (n = 12), and dyspnea (n = 9).

In evaluating the ranking based on the production amounts of HMPs, Uhwangchungsim-won secured the top position (4th in frequently reported adverse events), whereas Bangpungtongseong-san claimed the sixth spot (1st in frequently reported adverse events). Notably, 12 of the 15 most frequently reported HMPs were among the top 15 in the ranking of production amounts.

HMPs associated with serious adverse events, as detailed in above Section 3.3, include Eunkyo-san (24 cases), Bangpungtongseong-san (12 cases), Ssanghwa-tang (12 cases), Sogunjung-tang (9 cases), Kyeongok-go (8 cases), and Uhwangchungsim-won (7 cases). All six HMPs were consistently present in the top 15 most frequently reported HMPs associated with overall adverse events.

### 3.5 Causality assessment results

Among the 1,054 ICSRs, there were 2,884 instances in which the causality assessment results were available for each combination of HMP and adverse events. Among these, 1,099 were related to HMPs, whereas the remaining 1,785 pertained to concurrently administered medications other than HMPs. Causality assessment results and frequent HMP-adverse event combinations are presented in Table 7. Regarding HMP-related cases, the highest number of cases was categorized as possible (n = 616, 56.1%), followed by probable/likely (n = 161, 14.6%).

**TABLE 7:** Causality assessment results for 1,099 combinations of licensed herbal medicine products and adverse events.

Causality assessment	Cases, n (%)	Most frequent HMP–AE combination (n)
Certain	27 (2.5)	Jagyakgamcho-tang: constipation (3) Banggiwhanggi-tang: Dry mouth (2), Insomnia (2) Bangpungtongseong-san: Dry mouth (2)
Probable/likely	161 (14.6)	Eunkyo-san: Urticaria (7), Angioedema (5), Rash (4) Woohwangchungsim-won: Somnolence (4)
Possible	616 (56.1)	Bangpungtongseong-san: Insomnia (36) Kyuongok-go: Diarrhoea (19)
Unlikely	132 (12.0)	Bangpungtongseong-san: Dry mouth (8), Somnolence (4), Tremor (4), Dizziness (3), Sleep disorder (3) Galgeun-tang: Generalised oedema (3), Weight decreased (3)
Conditional/unclassified	19 (1.7)	Bojungikgi-tang: Weight increased (3) Kyuongok-go: Abdominal discomfort (2) Ssanghwa-tang: Diarrhoea (2)
Unassessable/unclassifiable	138 (12.6)	Kyuongok-go: Pyrexia (6), Diarrhoea (5), Erythema (5), Hypersensitivity (5), Rash (5), Flushing (4), Pruritus (4), Urticaria (4) Gongjin-dan: Pruritus (4)
Not applicable	6 (0.5)	Ssanghwa-tang: Product quality issue (2) Banhasasim-tang: Vertigo (1), Vomiting (1) Bangpungtongseong-san: Product prescribing error (1) Ssanghwa-tang: Expired product administered (1)

### 3.6 Critical adverse events: hepatobiliary, pulmonary, anaphylactic, and pseudoaldosteronism

Among the total reported 1,629 adverse events, we examined those related to hepatobiliary, pulmonary, anaphylactic, and pseudoaldosteronism separately (Table 8). In terms of hepatobiliary adverse events, 53 cases were identified and reported in 30 ICSRs. The most frequently reported events were increases in alanine aminotransferase and aspartate aminotransferase levels, accounting for 21 cases each, and were often reported concurrently in the same ICSR. Regarding pulmonary adverse events, 44 cases were identified and reported in 43 ICSRs. The most frequently reported events were dyspnea (28 cases) and cough (8 cases). Regarding anaphylactic responses, 9 events were reported in 9 ICSRs. No cases of pseudoaldosteronism were observed in this study.

**TABLE 8:** Hepatobiliary, pulmonary, and anaphylactic adverse events.

	System organ class	High level group term	Preferred term	N
Hepatobiliary - 30 ICSRs - 13 males, 16 females, 1 unspecified - age: 19–65 years (18 cases), ≥65 years (10 cases), (unspecified: 2 cases)	Investigations	Hepatobiliary investigations	Alanine aminotransferase increased	21
	Investigations	Hepatobiliary investigations	Aspartate aminotransferase increased	21
	Investigations	Hepatobiliary investigations	Liver function test abnormal	3
	Hepatobiliary disorders	Hepatic and hepatobiliary disorders	Hepatic function abnormal	2
	Investigations	Hepatobiliary investigations	Blood bilirubin increased	2
	Hepatobiliary disorders	Hepatic and hepatobiliary disorders	Hepatitis acute	1
	Hepatobiliary disorders	Hepatic and hepatobiliary disorders	Hepatitis toxic	1
	Hepatobiliary disorders	Hepatic and hepatobiliary disorders	Jaundice	1
	Investigations	Hepatobiliary investigations	Hepatic enzyme increased	1
Pulmonary - 43 ICSRs - 13 males, 30 females, 1 unspecified - age: 2–11 years (1 cases), 19–65 years (31 cases), ≥65 years (6 cases), (unspecified: 5 cases)	Respiratory, thoracic and mediastinal disorders	Respiratory disorders NEC	Dyspnoea	28
	Respiratory, thoracic and mediastinal disorders	Respiratory disorders NEC	Cough	8
	Respiratory, thoracic and mediastinal disorders	Respiratory disorders NEC	Respiratory distress	2
	Infections and infestations	Infections - pathogen unspecified	Pneumonia	1
	Respiratory, thoracic and mediastinal disorders	Bronchial disorders (excl neoplasms)	Wheezing	1
	Respiratory, thoracic and mediastinal disorders	Pleural disorders	Pleural effusion	1
	Respiratory, thoracic and mediastinal disorders	Respiratory disorders NEC	Irregular breathing	1
	Respiratory, thoracic and mediastinal disorders	Respiratory disorders NEC	Respiratory depression	1
	Respiratory, thoracic and mediastinal disorders	Respiratory disorders NEC	Sputum increased	1
Anaphylactic responses - 9 ICSRs - 3 males, 6 females - age: 19–65 years (8 cases), ≥65 years (1 cases)	Immune system disorders	Allergic conditions	Anaphylactic reaction	8
	Immune system disorders	Allergic conditions	Anaphylactic shock	1

## 4 Discussion

Descriptive analyses of adverse events associated with HMPs, as reported by the KAERS from 2012 to 2021, provide valuable insights into the safety profiles of these products. During the study period, 1,054 ICSRs were extracted, encompassing 1,629 adverse events associated with 84 HMPs. Considering that the total number of ICSRs related to pharmaceuticals reported by the KAERS during the same period was 2,457,727 (Ministry of Food and Drug Safety, 2023), the 1,054 ICSRs accounted for only 0.04% of the total. As of 2021, South Korea has 20,757 pharmaceutical products, with a total production amount of 16.23 billion USD. Among these, HMPs comprise 134 types and 645 items (3.1%), contributing 1.21 billion USD (0.7%) to the total production amount (Korea Pharmaceutical and Bio-Pharma Manufacturers Association, 2023). In this context, “types” refer to different herbal medicine formulas, such as Bangpungtongseong-san, Kyeongok-go, and Eunkyo-san, while “items” refer to individual HMP manufactured by pharmaceutical companies based on these herbal medicine formulas. When considering the proportion of HMPs in the overall pharmaceutical landscape, in terms of the number of items and production amount, the 0.04% figure for adverse events associated with HMPs, compared to total adverse events, was deemed a very low percentage.

In China, Japan, and Taiwan, where similar herbal medicines are used under TEAM, China accounts for 13.4% (Song et al., 2023), Japan for 0.5% (Ito, 2016), and Taiwan for 1.7% (Chang et al., 2021) of adverse events related to herbal medicines compared to overall pharmaceutical adverse events. Compared to other countries, the rate of HMP-related adverse events in Korea appears to be relatively low. Despite the use of similar herbal medicine formulas, variations in the healthcare environment and market size of herbal medicines may contribute to differences in the occurrence and reporting of HMP-related adverse events. Nevertheless, the notably low proportion of HMP-related adverse events in Korea warrants an in-depth investigation into underlying causes. Potential factors include a lack of awareness in reporting adverse events occurring after herbal medicine administration and the existence of distribution channels for herbal medicines that operate outside pharmacovigilance systems (Choudhury et al., 2023), which may lead to underreporting of adverse events.

In the analyzed 1,054 ICSRs, pharmacists were the primary original reporters (44.6%), followed by consumers (28.2%) and doctors (13.1%). This distribution differs from the overall ICSRs in Korea, where nurses constitute the majority of original reporters at 47.3% (Korea Institute of Drug Safety and Risk Management, 2018). With Korea’s 28 regional pharmacovigilance centers being hospital-centered (Kang et al., 2017), hospital nurses often play a significant role in reporting adverse events. In contrast, for HMPs commonly sold as over-the-counter medications, it is speculated that pharmacists or consumers represent a higher proportion of original reporters. In traditional Korean medicine hospitals and clinics, herbal medicines prepared at individual medical institutes are more frequently utilized (National Institute for Korean Medicine Development, 2023). These herbal medicines, which are prepared at local medical institutes outside the pharmacovigilance system, may contribute to a lower proportion of doctors and other medical professionals as the original reporters of ICSRs.

The demographics of patients who reported adverse events related to HMPs closely mirrored those of patients who used traditional Korean medicine. According to a 2016 survey on the utilization of traditional Korean medicine (National Institute for Korean Medicine Development, 2018), 71.3% of patients were aged 20%–64% and 21.1% were 65 years or older. Similarly, in this study, 76.9% of patients experiencing adverse events were aged 19%–64% and 20.3% were 65 years or older. In terms of sex, the distribution of patients reporting adverse events showed a slight shift, with 30.3% being male and 69.7% female. This aligns with the demographic distribution of traditional Korean medicine users, where 42.5% are male and 57.5% are female (National Institute for Korean Medicine Development, 2018). In the Taiwan study, there was a higher prevalence in the age groups of 36–65 years (56.4%) and 65 or older (20.8%), with a majority being females (66.7%) (Chang et al., 2021), which is consistent with the results of this study. This suggests that the middle-aged to older population, particularly females, who frequently use herbal medicine, also experience a significant number of adverse events related to herbal medicine.

The most frequently reported SOC was gastrointestinal disorders (28.7%), followed by skin and subcutaneous tissue disorders (20.1%). At the PT level, the highest frequencies were observed for diarrhea (5.8%), urticaria (5.3%), and pruritus (4.7%). According to the results of the disproportionality analysis, adverse events that were reported more frequently with HMPs compared to all other drugs included abdominal discomfort, insomnia, and palpitations. These findings suggest that certain adverse events, such as abdominal discomfort, insomnia, and palpitations, may be more specifically associated with HMPs compared to other pharmaceuticals. These findings, specific to herbal medicines, differ slightly from the overall pharmacovigilance data for all pharmaceuticals; as of 2017, the most frequent adverse events were nausea (16.3%), pruritus (11.0%), vomiting (7.6%), urticaria (7.5%), and dizziness (7.2%) (Korea Institute of Drug Safety and Risk Management, 2021). A study based on the Adverse Event Database in Taiwan (Chang et al., 2021) revealed similar results regarding herbal medicine-related adverse events, with gastrointestinal disorders (33.4%) and skin and subcutaneous tissue disorders (21.2%) being the most prevalent. Furthermore, analysis of the WHO database, VigiBase, revealed that ICSRs related to traditional medicines, particularly those associated with the SOC of skin and subcutaneous tissue disorders, constituted 19.9%. Related symptoms, including pruritus, rash, and urticaria, were consistent with those observed in our study (Barvaliya et al., 2023), underscoring the significance of skin and subcutaneous tissue disorders in herbal medicine-related adverse events.

Conversely, the frequency order of SOCs for adverse events related to HMPs reported in the Japanese Adverse Drug Event Report database differed from those in Korea and Taiwan. According to the Japanese database (Shimodaira et al., 2014), the most frequent SOCs were hepatobiliary disorders (34%); respiratory, thoracic and mediastinal disorders (26%); metabolism and nutrition disorders (9%); endocrine disorders (5%); and skin and subcutaneous tissue disorders (4%). Adverse events under hepatobiliary disorders were very rare in Korea (0.3%) and Taiwan (4.3%), while those under respiratory, thoracic, and mediastinal disorders were minimal in Korea (3.9%) and Taiwan (5.2%). Despite the use of herbal medicines similar to those used in TEAM, the types of reported adverse events exhibited distinct patterns. This may be attributed to the fact that adverse event reporting in Korea is primarily based on over-the-counter HMPs, whereas in Japan, adverse events are primarily reported for prescription drugs (Shimada et al., 2017; 2019). Most cases reported in Korea and Taiwan predominantly involved mild gastrointestinal and skin-related symptoms, whereas in Japan, there is a higher prevalence of hepatobiliary and pulmonary disorders.

Korea, China, Japan, and Taiwan use similar herbal medicines under the TEAM framework. We compared 20 HMPs with a high frequency of adverse event reports with results from Japan and Taiwan. Seven HMPs, Jakyakgamcho-tang, Bangpungtongsung-san, Galgeuntang, Banhasasim-tang, Bojungikgi-tang, Maekmundong-tang, and Socheongryong-tang, were commonly included in the Japanese data (Shimada et al., 2019). Four HMPs, Socheongryong-tang, Banhasasim-tang, Cheonwangbosimdan, and Galgeuntang, were commonly included in the Taiwanese data (Chang et al., 2021). Among these, three HMPs, Galgeuntang, Socheongryong-tang, and Banhasasim-tang, were commonly associated with a high frequency of adverse event reports in Korea, Taiwan, and Japan. It is not appropriate to conclude that these HMPs cause more adverse events than the other HMPs. Instead, it is reasonable to interpret that HMPs are commonly used across these regions, leading to a higher frequency of adverse event reports.

Among the 84 HMPs, Bangpungtongseong-san, which ranked sixth in terms of production amount, had the highest number of reported adverse events (297 cases). Analysis of data from the Japanese Adverse Event Reporting Database revealed that the most commonly reported herbal medicine was Bangpungtongseong-san (Bofu-tsusho-san in Japanese) (Ito, 2016; Shimada et al., 2019). Bangpungtongseong-san is one of the herbal medicines most frequently associated with adverse reactions in Korea and Japan. In a separate Japanese study, the estimated incidences of liver disorders related to Bangpungtongseong-san was 7.44 per 100,000 patients and that of pseudoaldosteronism was estimated to be 0.30 per 100,000 patients (Arai et al., 2020). Although commonly reported side effects of Bangpungtongseong-san include insomnia, abdominal discomfort, headache, and diarrhea, the prevalence of adverse hepatobiliary events is particularly noteworthy. An analysis of Japanese adverse event reporting data indicated a significant odds ratio for liver injury associated with Bangpungtongseong-san (Ishida et al., 2022). Therefore, when prescribing or consuming Bangpungtongseong-san, careful consideration of its potential side effects, particularly those related to hepatobiliary function, is crucial.

This study has several limitations. First, the analyzed data were based on voluntary reports of adverse events, introducing the possibility of underreporting. Furthermore, due to the nature of the spontaneous reporting system, we relied on the cases reported as “serious adverse events” in the ICSRS without modification, even if some of these events may not strictly meet the definition. Additionally, ICSRs had insufficient information to evaluate the causal relationship between HMPs and adverse reactions, and often lacked detailed information about the patients’ underlying diseases and their associated clinical manifestations. Therefore, the results of this study should be interpreted as preliminary and considered as suggestive rather than definitive information. Secondly, Korea’s adverse event reporting system focuses exclusively on pharmaceuticals authorized by the MFDS. Currently, there is no established system for reporting adverse events associated with herbal medicines prepared at local clinics or hospitals. Given the widespread use of both HMPs manufactured by pharmaceutical companies (28.5% utilization rate) and herbal medicines prepared at individual medical institutes (26.7% utilization rate) in Korea (National Institute for Korean Medicine Development, 2023), the KAERS database can only capture half of the adverse events associated with herbal medicines. This emphasizes the need for improvements in reporting systems to address these data coverage gaps. Third, there was a considerable amount of missing data in the ICSRs reports. For instance, there were notable gaps in the data related to the start and end dates of adverse events, actions taken concerning HMPs, and start and end dates of administration. To enhance the quality of adverse event reports related to herbal medicines, education on the importance of reporting, essential reporting elements, reporting methods, and methods for evaluating severity and causality is necessary (Kim et al., 2021a).

Despite the inherent limitations of relying on voluntary adverse reaction reports, this study provided useful insights into the landscape of herbal medicine-related adverse events in South Korea. Future research is required to estimate the incidence of adverse events and evaluate their causality. In addition, there is a pressing need to broaden the reporting system to include herbal medicines prepared by individual medical institutes.

## Data Availability

The data analyzed in this study were obtained from the Korea Institute of Drug Safety and Risk Management (KIDS KAERS DB, Data No. 2205A0042) and are not publicly available owing to the KIDS data protection policy. Requests to access the datasets should be directed to the KIDS (https://open.drugsafe.or.kr/).
