# Supplementary material for: Adverse events associated with herbal medicine products reported in the Korea Adverse Event Reporting System from 2012 to 2021
Source: Front Pharmacol. 2024 Oct 21;15:1378208. doi: 10.3389/fphar.2024.1378208 (PMC11532164; doi:10.3389/fphar.2024.1378208)
Supplement: Supplementary file 1 [file Table1.DOCX]

Supplementary Material

**Supplementary Table 1.** List of 84 licensed herbal medicine products

| **No** | **Licensed herbal medicine products** | | |
| --- | --- | --- | --- |
|  | Korean | Chinese | Japanese |
| 1 | Antae-eum | Antai-yin | - |
| 2 | Banggiwhanggi-tang | Fangjihuangqi-tang | Boiogi-to |
| 3 | Bangpungtongseong-san | Fangfengtongsheng-san | Bofutsusho-san |
| 4 | Banhabaekchulcheonma-tang | Banxiabaizhutianma-tang | Hangebyakujutsutemma-to |
| 5 | Banhahubag-tang | Banxiahoupu-tang | Hangekoboku-to |
| 6 | Banhasasim-tang | Banxiaxiexin-tang | Hangeshashin-to |
| 7 | Bojungikgi-tang | Buzhongyiqi-tang | Hochuekki-to |
| 8 | Buhnsimgi-eum | Fenxinqi-yin | Bunsinki-in |
| 9 | Bulhwangeumjeonggi-san | Buhuanjinzhengqi-san | Fukankinshoki-san |
| 10 | Chengsimyeunja-eum | Qingxinlianzi-yin | Seishinrenshi-in |
| 11 | Cheongsanggyeontong-tang | Qingshangjuantong-tang | Seijokentsu-to |
| 12 | Cheonwangbosim-dan | Tianwangbuxin-dan | Tennouhoshin-tan |
| 13 | Chongmyung-tang | Congming-tang | - |
| 14 | Daegunjoong-tang | Dajianzhong-tang | Daikenchu-to |
| 15 | Daehwajung-eum | Dawenzhong-yin | - |
| 16 | Daesiho-tang | Dachaihu-tang | Daisaiko-to |
| 17 | Daeyoung-jeon | Daying-jian | Taiei-sen |
| 18 | Danggwijakyak-san | Dangguishaoyao-san | Tokishakuyaku-san |
| 19 | Danggwisu-san | Dangguixu-san | Tokishu-san |
| 20 | Dokhwalgisaeng-tang | Duhuojisheng-tang | Dokkatsukisei-to |
| 21 | Eoggan-sangajinpiubanha | Yigan-sanjiachenpibanxia | Yokukan-sankachimpihange |
| 22 | Eunkyo-san | Yinqiao-san | Yinqiao-san |
| 23 | Galgeun-tang | Gegen-tang | Kakkon-to |
| 24 | Gamiondam-tang | Jiaweiwendan-tang | Kamiuntan-to |
| 25 | Gamisoyo-san | Jiaweixiaoyao-san | Kamishoyo-san |
| 26 | Goakhyangjeonggi-san | Huoxiangzhengqi-san | Kakkoshoki-san |
| 27 | Gongjin-dan | Gongchen-Dan | - |
| 28 | Gumiganghwal-tang | Jiuweiqianghuo-tang | Kumikyokatsu-to |
| 29 | Gungha-tang | Xiongxia-tang | - |
| 30 | Gyejibokryeong-hwan | Guizhifuling-wan | Keishibukuryo-gan |
| 31 | Gyeji-tang | Guizhi-tang | Keishi-to |
| 32 | Hwangryeonhaedok-tang | Huanglianjiedu-tang | Orengedoku-to |
| 33 | Hyangsapyeongwi-san | Xiangshapingwei-san | Koshaheii-san |
| 34 | Hyangsayukgunja-tang | Xiangshaliujunzi-tang | Kosharikkunshi-to |
| 35 | Hyangso-san | Xiangsu-san | Koso-san |
| 36 | Hyeonggaeyeongyo-tang | Jingjielianqiao-tang | Keigairengyo-to |
| 37 | Ijin-tang | Erchen-tang | Nichin-to |
| 38 | Ijung-tang | Lizhong-tang | Richu-to |
| 39 | Insampaedok-san | Renshenbaidu-san | Ninjinhaidoku-san |
| 40 | Insamyangyoung-tang | Renshenyangrong-tang | Ninjin’yoei-to |
| 41 | Jaeumganghwa-tang | Ziyinjianghuo-tang | Jiinkoka-to |
| 42 | Jagamcho-tang | Zhigancao-tang | Shakanzo-to |
| 43 | Jagyakgamcho-tang | Shaoyaogancao-tang | Shakuyakukanzo-to |
| 44 | Kyuongok-go | Qiongyu-gao | Keigyoku-kou |
| 45 | Maekmundong-tang | Maimendong-tang | Bakumondo-to |
| 46 | Mahwang-tang | Mahuang-tang | Mao-to |
| 47 | Majain-hwan | Maziren-wan | Mashinin-gan |
| 48 | Naeso-san | Neixiao-san | Naishou-san |
| 49 | Nokyongdaebo-tang | Lurongdabu-tang | - |
| 50 | Ojeog-san | Wuji-san | Goshaku-san |
| 51 | Onbak-won | Wenbai-yuan | - |
| 52 | Onchung-eum | Wenqing-yin | Unsei-in |
| 53 | Onkyeong-tang | Wenjing-tang | Unkei-to |
| 54 | Oryung-san | Wuling-san | Gorei-san |
| 55 | Oyaksungi-san | Wuyaoshunqi-san | Uyakujunki-san |
| 56 | Palmijihwang-hwan | Baweidihuang-tang | Hachimijio-gan |
| 57 | Palmul-tang | Bawu-tang | - |
| 58 | Pyeongwi-san | Pingwei-san | Heii-san |
| 59 | Saengmaek-san | Shengmai-san | Seimyaku-san |
| 60 | Sagunja-tang | Sizunji-tang | Shikunshi-to |
| 61 | Samchulgeonbi-tang | Shenzhujianpi-tang | Sanjutsukenhi-to |
| 62 | Samryeongbaekchul-san/ | Shenlingbaizhu-san | Jinryobyakujutsu-san |
| 63 | Samso-eum | Shensu-yin | Jinso-in |
| 64 | Samul-tang | Siwu-tang | Shimotsu-to |
| 65 | Sanjoin-tang | Suanzaoren-tang | Sansonin-to |
| 66 | Sibjeondaebo-tang | Shiquandabu-tang | Juzentaiho-to |
| 67 | Siryung-tang | Chailing-tang | Sairei-to |
| 68 | Socheongryong-tang | Xiaoqinglong-tang | Shoseiryu-to |
| 69 | Sogeonjung-tang | Xiaojianzhong-tang | Shokenchu-to |
| 70 | Sokyunghwalhyeol-tang / | Shujinghuoxue-tang | Sokeikakketsu-to |
| 71 | Sopung-san | Xiaofeng-san | Shofu-san |
| 72 | Sosiho-tang | Xiaochaihu-tang | Shosaiko-to |
| 73 | Ssanggum-tang | - | - |
| 74 | Ssanghwa-tang | Shuanghe-tang | Souwa-to |
| 75 | Takrisodok-eum | Tuolixiaodu-yin | Takurishodoku-in |
| 76 | Wiryeong-tang | Weiling-tang | Irei-to |
| 77 | Woohwangchungsim-won | Niuhuangqingxin-whan | Goouseishin-gen |
| 78 | Woohwangporyong-hwan | Niuhuangbaolong-wan | - |
| 79 | Yeonggyechulgam-tang / | Lingguizhugan-tang | Ryokeijutsukan-to |
| 80 | Yeongyopaedog-san | Lianqiaobaidu-san | - |
| 81 | Yongdamsagan-tang | Longdanxiegan-tang | Ryutanshakan-to |
| 82 | Youngseonjetong-eum | Lingxianqugou-yin | - |
| 83 | Yukgunja-tang | Liujunzi-tang | Rikkunshi-to |
| 84 | Yukmijihwang-tang | Liuweidihuang-tang | Rokumijio-to |

**Supplementary Table 2.** Frequency of adverse events classified by System Organ Class (SOC) and High Level Group Term (HLGT)

| SOC | HLGT | n |
| --- | --- | --- |
| Gastrointestinal disorders | Dental and gingival conditions | 1 |
|  | Diverticular disorders | 1 |
|  | Gastrointestinal conditions NEC | 4 |
|  | Gastrointestinal haemorrhages NEC | 4 |
|  | Gastrointestinal inflammatory conditions | 4 |
|  | Gastrointestinal motility and defaecation conditions | 126 |
|  | Gastrointestinal signs and symptoms | 283 |
|  | Gastrointestinal ulceration and perforation | 2 |
|  | Malabsorption conditions | 1 |
|  | Oral soft tissue conditions | 5 |
|  | Salivary gland conditions | 32 |
|  | Tongue conditions | 4 |
| Skin and subcutaneous tissue disorders | Angioedema and urticaria | 109 |
|  | Cornification and dystrophic skin disorders | 2 |
|  | Epidermal and dermal conditions | 192 |
|  | Skin appendage conditions | 22 |
|  | Skin vascular abnormalities | 2 |
| Nervous system disorders | Central nervous system vascular disorders | 2 |
|  | Cranial nerve disorders (excl neoplasms) | 2 |
|  | Headaches | 52 |
|  | Mental impairment disorders | 1 |
|  | Movement disorders (incl parkinsonism) | 14 |
|  | Neurological disorders NEC | 110 |
|  | Neuromuscular disorders | 2 |
|  | Peripheral neuropathies | 1 |
|  | Seizures (incl subtypes) | 1 |
| General disorders and administration site conditions | Administration site reactions | 3 |
|  | Body temperature conditions | 21 |
|  | Fatal outcomes | 1 |
|  | General system disorders NEC | 115 |
|  | Therapeutic and nontherapeutic effects (excl toxicity) | 6 |
| Psychiatric disorders | Anxiety disorders and symptoms | 2 |
|  | Changes in physical activity | 2 |
|  | Depressed mood disorders and disturbances | 3 |
|  | Disturbances in thinking and perception | 3 |
|  | Mood disorders and disturbances NEC | 14 |
|  | Psychiatric and behavioural symptoms NEC | 1 |
|  | Psychiatric disorders NEC | 1 |
|  | Sexual dysfunctions, disturbances and gender identity disorders | 1 |
|  | Sleep disorders and disturbances | 83 |
| Investigations | Cardiac and vascular investigations (excl enzyme tests) | 9 |
|  | Hepatobiliary investigations | 48 |
|  | Metabolic, nutritional and blood gas investigations | 4 |
|  | Neurological, special senses and psychiatric investigations | 2 |
|  | Physical examination and organ system status topics | 15 |
|  | Renal and urinary tract investigations and urinalyses | 3 |
| Respiratory, thoracic and mediastinal disorders | Bronchial disorders (excl neoplasms) | 1 |
|  | Pleural disorders | 1 |
|  | Respiratory disorders NEC | 41 |
|  | Respiratory tract signs and symptoms | 9 |
|  | Upper respiratory tract disorders (excl infections) | 12 |
| Cardiac disorders | Cardiac arrhythmias | 6 |
|  | Cardiac disorders, signs and symptoms NEC | 37 |
|  | Myocardial disorders | 1 |
| Eye disorders | Anterior eye structural change, deposit and degeneration | 1 |
|  | Eye disorders NEC | 19 |
|  | Glaucoma and ocular hypertension | 1 |
|  | Ocular infections, irritations and inflammations | 6 |
|  | Ocular structural change, deposit and degeneration NEC | 5 |
|  | Vision disorders | 3 |
| Musculoskeletal and connective tissue disorders | Bone disorders (excl congenital and fractures) | 1 |
|  | Joint disorders | 5 |
|  | Muscle disorders | 13 |
|  | Musculoskeletal and connective tissue deformities (incl intervertebral disc disorders) | 3 |
|  | Musculoskeletal and connective tissue disorders NEC | 10 |
| Infections and infestations | Fungal infectious disorders | 1 |
|  | Infections - pathogen unspecified | 22 |
| Vascular disorders | Decreased and nonspecific blood pressure disorders and shock | 7 |
|  | Vascular disorders NEC | 15 |
|  | Vascular hypertensive disorders | 1 |
| Immune system disorders | Allergic conditions | 19 |
| Injury, poisoning and procedural complications | Injuries by physical agents | 1 |
|  | Injuries NEC | 5 |
|  | Medication errors and other product use errors and issues | 11 |
|  | Off label uses and intentional product misuses/use issues | 1 |
| Metabolism and nutrition disorders | Appetite and general nutritional disorders | 13 |
|  | Electrolyte and fluid balance conditions | 3 |
|  | Vitamin related disorders | 1 |
| Renal and urinary disorders | Renal disorders (excl nephropathies) | 2 |
|  | Urinary tract signs and symptoms | 11 |
| Reproductive system and breast disorders | Breast disorders | 4 |
|  | Male reproductive tract infections and inflammations | 1 |
|  | Menstrual cycle and uterine bleeding disorders | 2 |
|  | Vulvovaginal disorders (excl infections and inflammations) | 1 |
| Ear and labyrinth disorders | Aural disorders NEC | 2 |
|  | Inner ear and VIIIth cranial nerve disorders | 4 |
| Hepatobiliary disorders | Hepatic and hepatobiliary disorders | 5 |
| Blood and lymphatic system disorders | Anaemias nonhaemolytic and marrow depression | 1 |
|  | Coagulopathies and bleeding diatheses (excl thrombocytopenic) | 1 |
|  | White blood cell disorders | 1 |
| Product issues | Product quality, supply, distribution, manufacturing and quality system issues | 3 |

**Supplementary Table 3.** Frequency of adverse events reported as serious adverse events

| **System Organ Class** | **High Level Group Term** | **Preferred Term** | **N** |
| --- | --- | --- | --- |
| **Death** | | | |
| Gastrointestinal disorders | Gastrointestinal motility and defaecation conditions | Diarrhoea | 1 |
| Gastrointestinal disorders | Gastrointestinal signs and symptoms | Vomiting | 1 |
| General disorders and administration site conditions | Body temperature conditions | Pyrexia | 1 |
| General disorders and administration site conditions | Fatal outcomes | Death | 1 |
| General disorders and administration site conditions | General system disorders NEC | Multiple organ dysfunction syndrome | 1 |
| Hepatobiliary disorders | Hepatic and hepatobiliary disorders | Hepatitis toxic | 1 |
| Infections and infestations | Infections - pathogen unspecified | Pneumonia | 1 |
| Infections and infestations | Infections - pathogen unspecified | Septic shock | 1 |
| **Life-threatening** | | | |
| Gastrointestinal disorders | Gastrointestinal signs and symptoms | Nausea | 1 |
| Nervous system disorders | Neurological disorders NEC | Dizziness | 1 |
| Respiratory, thoracic and mediastinal disorders | Respiratory disorders NEC | Dyspnoea | 1 |
| **Hospitalization (initial or prolonged)** | | | |
| Investigations | Hepatobiliary investigations | Alanine aminotransferase increased | 7 |
| Investigations | Hepatobiliary investigations | Aspartate aminotransferase increased | 7 |
| Respiratory, thoracic and mediastinal disorders | Respiratory disorders NEC | Dyspnoea | 3 |
| Gastrointestinal disorders | Gastrointestinal signs and symptoms | Nausea | 2 |
| Investigations | Hepatobiliary investigations | Liver function test abnormal | 2 |
| Skin and subcutaneous tissue disorders | Epidermal and dermal conditions | Rash | 2 |
| Blood and lymphatic system disorders | White blood cell disorders | Febrile neutropenia | 1 |
| Gastrointestinal disorders | Gastrointestinal signs and symptoms | Abdominal pain | 1 |
| Gastrointestinal disorders | Gastrointestinal ulceration and perforation | Gastric ulcer | 1 |
| Gastrointestinal disorders | Gastrointestinal ulceration and perforation | Intestinal ulcer | 1 |
| General disorders and administration site conditions | Body temperature conditions | Pyrexia | 1 |
| General disorders and administration site conditions | General system disorders NEC | Asthenia | 1 |
| General disorders and administration site conditions | General system disorders NEC | Condition aggravated | 1 |
| General disorders and administration site conditions | General system disorders NEC | Face oedema | 1 |
| Hepatobiliary disorders | Hepatic and hepatobiliary disorders | Hepatitis acute | 1 |
| Hepatobiliary disorders | Hepatic and hepatobiliary disorders | Jaundice | 1 |
| Immune system disorders | Allergic conditions | Anaphylactic shock | 1 |
| Infections and infestations | Infections - pathogen unspecified | Anal abscess | 1 |
| Infections and infestations | Infections - pathogen unspecified | Rash pustular | 1 |
| Injury, poisoning and procedural complications | Injuries NEC | Fall | 1 |
| Investigations | Hepatobiliary investigations | Blood bilirubin increased | 1 |
| Metabolism and nutrition disorders | Appetite and general nutritional disorders | Cachexia | 1 |
| Metabolism and nutrition disorders | Electrolyte and fluid balance conditions | Hyponatraemia | 1 |
| Musculoskeletal and connective tissue disorders | Bone disorders (excl congenital and fractures) | Bone pain | 1 |
| Musculoskeletal and connective tissue disorders | Muscle disorders | Myalgia | 1 |
| Musculoskeletal and connective tissue disorders | Musculoskeletal and connective tissue disorders NEC | Pain in extremity | 1 |
| Nervous system disorders | Neurological disorders NEC | Dizziness | 1 |
| Respiratory, thoracic and mediastinal disorders | Pleural disorders | Pleural effusion | 1 |
| Skin and subcutaneous tissue disorders | Epidermal and dermal conditions | Dermatitis | 1 |
| Skin and subcutaneous tissue disorders | Epidermal and dermal conditions | Dermatitis bullous | 1 |
| Skin and subcutaneous tissue disorders | Epidermal and dermal conditions | Pruritus | 1 |
| Skin and subcutaneous tissue disorders | Epidermal and dermal conditions | Rash pruritic | 1 |
| Skin and subcutaneous tissue disorders | Epidermal and dermal conditions | Stevens-Johnson syndrome | 1 |
| Vascular disorders | Decreased and nonspecific blood pressure disorders and shock | Shock | 1 |
| **Important medical events** | | | |
| Respiratory, thoracic and mediastinal disorders | Respiratory disorders NEC | Dyspnoea | 5 |
| Skin and subcutaneous tissue disorders | Angioedema and urticaria | Angioedema | 4 |
| Skin and subcutaneous tissue disorders | Angioedema and urticaria | Urticaria | 4 |
| Immune system disorders | Allergic conditions | Anaphylactic reaction | 3 |
| General disorders and administration site conditions | General system disorders NEC | Chest discomfort | 2 |
| Nervous system disorders | Neurological disorders NEC | Dizziness | 2 |
| Cardiac disorders | Cardiac arrhythmias | Tachycardia | 1 |
| Eye disorders | Eye disorders NEC | Periorbital oedema | 1 |
| Eye disorders | Glaucoma and ocular hypertension | Angle closure glaucoma | 1 |
| Eye disorders | Ocular structural change, deposit and degeneration NEC | Orbital oedema | 1 |
| Eye disorders | Vision disorders | Vision blurred | 1 |
| Gastrointestinal disorders | Gastrointestinal signs and symptoms | Retching | 1 |
| Gastrointestinal disorders | Gastrointestinal ulceration and perforation | Gastric ulcer | 1 |
| Gastrointestinal disorders | Gastrointestinal ulceration and perforation | Intestinal ulcer | 1 |
| Gastrointestinal disorders | Salivary gland conditions | Dry mouth | 1 |
| General disorders and administration site conditions | General system disorders NEC | Face oedema | 1 |
| Infections and infestations | Infections - pathogen unspecified | Anal abscess | 1 |
| Nervous system disorders | Headaches | Headache | 1 |
| Nervous system disorders | Movement disorders (incl parkinsonism) | Paralysis | 1 |
| Psychiatric disorders | Disturbances in thinking and perception | Hallucination | 1 |
| Psychiatric disorders | Disturbances in thinking and perception | Hallucination, auditory | 1 |
| Respiratory, thoracic and mediastinal disorders | Respiratory disorders NEC | Respiratory distress | 1 |
| Respiratory, thoracic and mediastinal disorders | Upper respiratory tract disorders (excl infections) | Nasal mucosal hypertrophy | 1 |
| Respiratory, thoracic and mediastinal disorders | Upper respiratory tract disorders (excl infections) | Nasal oedema | 1 |
| Skin and subcutaneous tissue disorders | Epidermal and dermal conditions | Blister | 1 |
| Skin and subcutaneous tissue disorders | Epidermal and dermal conditions | Pruritus | 1 |
| Skin and subcutaneous tissue disorders | Epidermal and dermal conditions | Rash | 1 |
| Skin and subcutaneous tissue disorders | Epidermal and dermal conditions | Rash pruritic | 1 |
| Skin and subcutaneous tissue disorders | Epidermal and dermal conditions | Stevens-Johnson syndrome | 1 |

**Supplementary Table 4.** Demographics of safety reports comparing serious adverse events versus not serious adverse events.

|  | **SAE** | **Non-SAE** | **Odds ratio (95% CI)** |
| --- | --- | --- | --- |
| **Age at the time of occurrence** |  |  |  |
| –64 years | 36 | 582 | Reference |
| 65 years ~ | 9 | 148 | 0.98 (0.46, 2.09) |
| **Sex** |  |  |  |
| Male | 17 | 254 | Reference |
| Female | 31 | 592 | 0.78 (0.43, 1.44) |

**Supplementary Table 5.** The compositions of 15 herbal medicine products

| **Herbal medicine product name** | **Compositional drug name** | **Compositional drug species name** | **Part used** | **Dosage (g)** |
| --- | --- | --- | --- | --- |
| Bangpungtongseong-san | Talcum | - | - | 1.67 |
|  | Glycyrrhizae Radix et Rhizoma | Glycyrrhiza uralensis Fisch. ex DC. [Fabaceae] | Root and rhizome | 0.67 |
|  | Gypsum | - | - | 1 |
|  | Scutellariae Radix | Scutellaria baicalensis Georgi [Lamiaceae] | Root | 0.67 |
|  | Platycodonis Radix | Platycodon grandiflorus (Jacq.) A.DC. [Campanulaceae] | Root | 0.67 |
|  | Saposhnikoviae Radix | Saposhnikovia divaricata (Turcz.) Schischk. [Apiaceae] | Root | 0.4 |
|  | Paeoniae Radix | Paeonia lactiflora Pall. [Paeoniaceae] | Root | 0.4 |
|  | Cnidii Rhizoma | Ligusticum officinale (Makino) Kitag. [Apiaceae] | Rhizome | 0.4 |
|  | Angelicae Gigantis Radix | Angelica gigas Nakai [Apiaceae] | Root | 0.4 |
|  | Rhei Radix et Rhizoma | Rheum undulatum L. [Polygonaceae] | Root and rhizome | 0.5 |
|  | Ephedrae Herba | Ephedra sinica Stapf [Ephedraceae] | Stem | 0.4 |
|  | Mentha arvensis | Mentha arvensis L. [Lamiaceae] | Aerial part | 0.4 |
|  | Forsythiae Fructus | Forsythia viridissima Lindl. [Oleaceae] | Fruit | 0.4 |
|  | Natrii sulfas | - | - | 0.5 |
|  | Schizonepetae Spica | Nepeta tenuifolia Benth. [Lamiaceae] | Flower stalk | 0.4 |
|  | Atractylodis Rhizoma Alba | Atractylodes lancea (Thunb.) DC. [Asteraceae] | Rhizome | 0.67 |
|  | Gardenia jasminoides | Gardenia jasminoides J. Ellis [Rubiaceae] | Fruit | 0.4 |
|  | Zingiberis Rhizoma Recens | Zingiber officinale Roscoe [Zingiberaceae] | Rhizome | 0.4 |
| Kyeongok-go | Rehmanniae Radix Recens | Rehmannia glutinosa (Gaertn.) DC. [Orobanchaceae] | Root | 39.9 |
|  | Poria Sclerotium | Poria cocos Wolf | Sclerotium | 12.4 |
|  | Ginseng Radix | Panax ginseng C.A.Mey. [Araliaceae] | Root | 6.2 |
|  | Honey | Apis cerana Fabricius | - | 41.5 |
| Eunkyo-san | Forsythiae Fructus | Forsythia viridissima Lindl. [Oleaceae] | Fruit | 1.42 |
|  | Lonicerae Flos | Lonicera japonica Thunb. [Caprifoliaceae] | Flower | 1.42 |
|  | Platycodonis Radix | Platycodon grandiflorus (Jacq.) A.DC. [Campanulaceae] | Root | 0.852 |
|  | Mentha arvensis | Mentha arvensis L. [Lamiaceae] | Aerial part | 0.852 |
|  | Lophatheri Herba | Lophatherum gracile Brongn. [Poaceae] | Aerial part | 0.568 |
|  | Glycyrrhizae Radix et Rhizoma | Glycyrrhiza uralensis Fisch. ex DC. [Fabaceae] | Root and rhizome | 0.852 |
|  | Schizonepetae Spica | Nepeta tenuifolia Benth. [Lamiaceae] | Flower stalk | 0.568 |
|  | Glycine Semen Preparata | Glycine max (L.) Merr. [Fabaceae] | Seed | 0.712 |
|  | Arctii Fructus | Arctium lappa L. [Asteraceae] | Fruit | 0.712 |
|  | Gazellae seu Saigae Cornu | Saiga tatarica | Horn | 0.044 |
| Uhwangchungsim-won | Dioscoreae Rhizoma | Dioscorea oppositifolia L. [Dioscoreaceae] | Rhizome | 0.263 |
|  | Glycyrrhizae Radix et Rhizoma | Glycyrrhiza uralensis Fisch. ex DC. [Fabaceae] | Root and rhizome | 0.188 |
|  | Ginseng Radix | Panax ginseng C.A.Mey. [Araliaceae] | Root | 0.094 |
|  | Typhae Pollen | Typha angustifolia L. [Typhaceae] | Pollen | 0.094 |
|  | Massa Medicata Fermentata | - | NA | 0.094 |
|  | Glycine Semen Germinatum | Glycine max (L.) Merr. [Fabaceae] | Seed | 0.066 |
|  | Cinnamomi Cortex | Cinnamomum verum J.Presl [Lauraceae] | Bark | 0.066 |
|  | Asini Corii Colla | Equus africanus asinus Linnaeus | Dry glue pieces made from the hide by stewing | 0.066 |
|  | Paeoniae Radix | Paeonia lactiflora Pall. [Paeoniaceae] | Root | 0.056 |
|  | Liriopis seu Ophiopogonis Tuber | Ophiopogon japonicus (Thunb.) Ker Gawl. [Asparagaceae] | Tuber | 0.056 |
|  | Scutellariae Radix | Scutellaria baicalensis Georgi [Lamiaceae] | Root | 0.056 |
|  | Angelicae Gigantis Radix | Angelica gigas Nakai [Apiaceae] | Root | 0.056 |
|  | Saposhnikoviae Radix | Saposhnikovia divaricata (Turcz.) Schischk. [Apiaceae] | Root | 0.056 |
|  | Atractylodis Rhizoma Alba | Atractylodes lancea (Thunb.) DC. [Asteraceae] | Rhizome | 0.056 |
|  | Bupleuri Radix | Bupleurum chinense DC. [Apiaceae] | Root | 0.047 |
|  | Platycodonis Radix | Platycodon grandiflorus (Jacq.) A.DC. [Campanulaceae] | Root | 0.047 |
|  | Armeniacae Semen | Prunus armeniaca L. [Rosaceae] | Seed | 0.047 |
|  | Poria Sclerotium | Poria cocos Wolf | Sclerotium | 0.047 |
|  | Cnidii Rhizoma | Ligusticum officinale (Makino) Kitag. [Apiaceae] | Rhizome | 0.047 |
|  | Bovis Calculus | Bos taurus Linnaeus | Dry gallstones | 0.045 |
|  | Gazellae seu Saigae Cornu | Saiga tatarica | Horn | 0.038 |
|  | Moschus | Moschus berezovskii Flerov | Dry secretions | 0.038 |
|  | Bomeolum | - | - | 0.008 |
|  | Ampelopsis Radix | Ampelopsis japonica (Thunb.) Makino [Vitaceae] | Root | 0.028 |
|  | Zingiberis Rhizoma Siccus | Zingiber officinale Roscoe [Zingiberaceae] | Rhizome | 0.028 |
| Galgeun-tang | Puerariae Radix | Pueraria montana var. lobata (Willd.) [Fabaceae] | Root | 2.67 |
|  | Ephedrae Herba | Ephedra sinica Stapf [Ephedraceae] | Stem | 1.33 |
|  | Cinnamomi Ramulus | Neolitsea cassia (L.) Kosterm. [Lauraceae] | Young branch | 1 |
|  | Paeoniae Radix | Paeonia lactiflora Pall. [Paeoniaceae] | Root | 1 |
|  | Glycyrrhizae Radix et Rhizoma | Glycyrrhiza uralensis Fisch. ex DC. [Fabaceae] | Root and rhizome | 0.67 |
|  | Zingiberis Rhizoma Siccus | Zingiber officinale Roscoe [Zingiberaceae] | Rhizome | 0.33 |
|  | Zizyphi Fructus | Ziziphus jujuba Mill. [Rhamnaceae] | Fruit | 1.33 |
| Ssanghwa-tang | Paeoniae Radix | Paeonia lactiflora Pall. [Paeoniaceae] | Root | 3.13 |
|  | Rehmanniae Radix Preparata | Rehmannia glutinosa (Gaertn.) DC. [Orobanchaceae] | Root | 1.25 |
|  | Astragali Radix | Astragalus mongholicus Bunge [Fabaceae] | Root | 1.25 |
|  | Angelicae Gigantis Radix | Angelica gigas Nakai [Apiaceae] | Root | 1.25 |
|  | Cnidii Rhizoma | Ligusticum officinale (Makino) Kitag. [Apiaceae] | Rhizome | 1.25 |
|  | Cinnamomi Cortex | Cinnamomum verum J.Presl [Lauraceae] | Bark | 0.94 |
|  | Zizyphi Fructus | Ziziphus jujuba Mill. [Rhamnaceae] | Fruit | 0.67 |
|  | Zingiberis Rhizoma Siccus | Zingiber officinale Roscoe [Zingiberaceae] | Rhizome | 0.5 |
|  | Glycyrrhizae Radix et Rhizoma | Glycyrrhiza uralensis Fisch. ex DC. [Fabaceae] | Root and rhizome | 0.94 |
| Bangkeehwangkee-tang | Sinomeni Caulis et Rhizoma | Sinomenium acutum (Thunb.) Rehder & E.H.Wilson [Menispermaceae] | Rhizome | 1.67 |
|  | Astragali Radix | Astragalus mongholicus Bunge [Fabaceae] | Root | 1.67 |
|  | Atractylodis Rhizoma Alba | Atractylodes lancea (Thunb.) DC. [Asteraceae] | Rhizome | 1 |
|  | Zingiberis Rhizoma Recens | Zingiber officinale Roscoe [Zingiberaceae] | Rhizome | 1 |
|  | Zizyphi Fructus | Ziziphus jujuba Mill. [Rhamnaceae] | Fruit | 1.33 |
|  | Glycyrrhizae Radix et Rhizoma | Glycyrrhiza uralensis Fisch. ex DC. [Fabaceae] | Root and rhizome | 0.67 |
| Cheonwangbosim-dan | Rehmanniae Radix Recens | Rehmannia glutinosa (Gaertn.) DC. [Orobanchaceae] | Root | 0.5 |
|  | Coptidis Rhizoma | Coptis chinensis Franch. [Ranunculaceae] | Rhizome | 0.25 |
|  | Ginseng Radix | Panax ginseng C.A.Mey. [Araliaceae] | Root | 0.625 |
|  | Angelicae Gigantis Radix | Angelica gigas Nakai [Apiaceae] | Root | 0.125 |
|  | Schisandrae Fructus | Schisandra chinensis (Turcz.) Baill. [Schisandraceae] | Fruit | 0.125 |
|  | Asparagi Tuber | Asparagus cochinchinensis (Lour.) Merr. [Asparagaceae] | Tuber | 0.125 |
|  | Liriopis seu Ophiopogonis Tuber | Ophiopogon japonicus (Thunb.) Ker Gawl. [Asparagaceae] | Tuber | 0.125 |
|  | Zizyphi Semen | Ziziphus jujuba Mill. [Rhamnaceae] | Seed | 0.125 |
|  | Thujae Semen | Platycladus orientalis (L.) Franco [Cupressaceae] | Seed | 0.125 |
|  | Scrophulariae Radix | Scrophularia ningpoensis Hemsl. [Scrophulariaceae] | Root | 0.0625 |
|  | Poria Sclerotium | Poria cocos Wolf | Sclerotium | 0.0625 |
|  | Salviae Miltiorrhizae Radix | Salvia miltiorrhiza Bunge [Lamiaceae] | Root | 0.0625 |
|  | Platycodonis Radix | Platycodon grandiflorus (Jacq.) A.DC. [Campanulaceae] | Root | 0.0625 |
|  | Polygalae Radix | Polygala senega L. [Polygalaceae] | Root | 0.0625 |
| Gongjin-dan | Cervi Parvum Cornu | Cervus nippon Temminck | Young horn | 1.333 |
|  | Angelicae Gigantis Radix | Angelica gigas Nakai [Apiaceae] | Root | 1.333 |
|  | Corni Fructus | Cornus officinalis Siebold & Zucc. [Cornaceae] | Fruit | 1.333 |
|  | Moschus | Moschus berezovskii Flerov | Dry secretions | 0.222 |
|  | Ginseng Radix | Panax ginseng C.A.Mey. [Araliaceae] | Root | 1.333 |
|  | Rehmanniae Radix Preparata | Rehmannia glutinosa (Gaertn.) DC. [Orobanchaceae] | Root | 1.333 |
| Socheongryong-tang | Ephedrae Herba | Ephedra sinica Stapf [Ephedraceae] | Stem | 1 |
|  | Paeoniae Radix | Paeonia lactiflora Pall. [Paeoniaceae] | Root | 1 |
|  | Asiasari Radix et Rhizoma | Asarum heterotropoides F.Schmidt [Aristolochiaceae] | Root and rhizome | 1 |
|  | Zingiberis Rhizoma Siccus | Zingiber officinale Roscoe [Zingiberaceae] | Rhizome | 1 |
|  | Glycyrrhizae Radix et Rhizoma | Glycyrrhiza uralensis Fisch. ex DC. [Fabaceae] | Root and rhizome | 1 |
|  | Cinnamomi Ramulus | Neolitsea cassia (L.) Kosterm. [Lauraceae] | Young branch | 1 |
|  | Pinelliae Tuber | Pinellia ternata (Thunb.) Makino [Araceae] | Tuber | 2 |
|  | Schisandrae Fructus | Schisandra chinensis (Turcz.) Baill. [Schisandraceae] | Fruit | 1 |
| Banhasasim-tang | Pinelliae Tuber | Pinellia ternata (Thunb.) Makino [Araceae] | Tuber | 1.67 |
|  | Scutellariae Radix | Scutellaria baicalensis Georgi [Lamiaceae] | Root | 1 |
|  | Ginseng Radix | Panax ginseng C.A.Mey. [Araliaceae] | Root | 1 |
|  | Glycyrrhizae Radix et Rhizoma | Glycyrrhiza uralensis Fisch. ex DC. [Fabaceae] | Root and rhizome | 1 |
|  | Zingiberis Rhizoma Siccus | Zingiber officinale Roscoe [Zingiberaceae] | Rhizome | 0.83 |
|  | Coptidis Rhizoma | Coptis chinensis Franch. [Ranunculaceae] | Rhizome | 0.33 |
|  | Zizyphi Fructus | Ziziphus jujuba Mill. [Rhamnaceae] | Fruit | 1 |
| Jakyak gamcho-tang | Paeoniae Radix | Paeonia lactiflora Pall. [Paeoniaceae] | Root | 2 |
|  | Glycyrrhizae Radix et Rhizoma | Glycyrrhiza uralensis Fisch. ex DC. [Fabaceae] | Root and rhizome | 2 |
| Ojeok-san | Atractylodis Rhizoma | Atractylodes lancea (Thunb.) DC. [Asteraceae] | Rhizome | 1.33 |
|  | Ephedrae Herba | Ephedra sinica Stapf [Ephedraceae] | Stem | 0.67 |
|  | Citri Unshius Pericarpium | Citrus reticulata Blanco [Rutaceae] | Pericarpium | 0.67 |
|  | Magnoliae Cortex | Magnolia officinalis Rehder & E.H.Wilson [Magnoliaceae] | Bark | 0.67 |
|  | Platycodonis Radix | Platycodon grandiflorus (Jacq.) A.DC. [Campanulaceae] | Root | 0.67 |
|  | Ponciri Fructus Immaturus | Citrus trifoliata L. [Rutaceae] | Fruit | 0.67 |
|  | Angelicae Gigantis Radix | Angelica gigas Nakai [Apiaceae] | Root | 0.67 |
|  | Zingiberis Rhizoma Siccus | Zingiber officinale Roscoe [Zingiberaceae] | Rhizome | 0.67 |
|  | Paeoniae Radix | Paeonia lactiflora Pall. [Paeoniaceae] | Root | 0.67 |
|  | Poria Sclerotium | Poria cocos Wolf | Sclerotium | 0.67 |
|  | Angelicae Dahuricae Radix | Angelica dahurica (Hoffm.) Benth. & Hook.f. ex Franch. & Sav. [Apiaceae] | Root | 0.67 |
|  | Cnidii Rhizoma | Ligusticum officinale (Makino) Kitag. [Apiaceae] | Rhizome | 0.67 |
|  | Pinelliae Tuber | Pinellia ternata (Thunb.) Makino [Araceae] | Tuber | 0.67 |
|  | Cinnamomi Ramulus | Neolitsea cassia (L.) Kosterm. [Lauraceae] | Young branch | 0.67 |
|  | Glycyrrhizae Radix et Rhizoma | Glycyrrhiza uralensis Fisch. ex DC. [Fabaceae] | Root and rhizome | 0.67 |
|  | Zizyphi Fructus | Ziziphus jujuba Mill. [Rhamnaceae] | Fruit | 0.67 |
|  | Cyperi Rhizoma | Cyperus rotundus L. [Cyperaceae] | Rhizome | 0.4 |
| Sogunjung-tang | Paeoniae Radix | Paeonia lactiflora Pall. [Paeoniaceae] | Root | 2 |
|  | Cinnamomi Ramulus | Neolitsea cassia (L.) Kosterm. [Lauraceae] | Young branch | 1.33 |
|  | Glycyrrhizae Radix et Rhizoma | Glycyrrhiza uralensis Fisch. ex DC. [Fabaceae] | Root and rhizome | 1 |
|  | Zingiberis Rhizoma Recens | Zingiber officinale Roscoe [Zingiberaceae] | Rhizome | 1.33 |
|  | Zizyphi Fructus | Ziziphus jujuba Mill. [Rhamnaceae] | Fruit | 1.33 |
|  | Oryzae Gluten | - | - | 6.67 |
| Maekmundong-tang | Liriopis seu Ophiopogonis Tuber | Ophiopogon japonicus (Thunb.) Ker Gawl. [Asparagaceae] | Tuber | 3.33 |
|  | Pinelliae Tuber | Pinellia ternata (Thunb.) Makino [Araceae] | Tuber | 1.67 |
|  | Ginseng Radix | Panax ginseng C.A.Mey. [Araliaceae] | Root | 0.67 |
|  | Glycyrrhizae Radix et Rhizoma | Glycyrrhiza uralensis Fisch. ex DC. [Fabaceae] | Root and rhizome | 0.67 |
|  | Oryzae Semen | Oryza sativa L. [Poaceae] | Seed | 3.33 |
|  | Zizyphi Fructus | Ziziphus jujuba Mill. [Rhamnaceae] | Fruit | 1 |
